# Influence of Homogenization and Solution Treatments Time on the Microstructure and Hardness of Inconel 718 Fabricated by Laser Powder Bed Fusion Process

**DOI:** 10.3390/ma13112574

**Published:** 2020-06-05

**Authors:** Eslam M. Fayed, Davood Shahriari, Mohammad Saadati, Vladimir Brailovski, Mohammad Jahazi, Mamoun Medraj

**Affiliations:** 1 Department of Mechanical, Industrial and Aerospace Engineering, Concordia University, 15151 Rue Sainte Catherine West, Montreal, QC H3G 2W1, Canada; e_fayed@encs.concordia.ca; 2 Department of Mechanical Engineering, École de Technologie Supérieure, 1100, Notre-Dame Street West, Montreal, QC H3C 1K3, Canada; davood.shahriari@etsmtl.ca (D.S.); mohammad.saadati.1@ens.etsmtl.ca (M.S.); vladimir.brailovski@etsmtl.ca (V.B.); mohammad.jahazi@etsmtl.ca (M.J.)

**Keywords:** additive manufacturing, laser powder bed fusion, metal 3D printing, nickel-based superalloy, IN718, microstructure, heat-treatment

## Abstract

In the present study, Inconel 718 (IN718) superalloy fabricated by laser powder bed fusion (LPBF) has been characterized focusing on the effect of both homogenization and solution treatment time on grains structure, crystallographic texture, precipitates formation/dissolution and material hardness. For this purpose, a heat-treatment time window with a wide range of soaking times for both treatments was established aiming to develop the optimal post-treatment conditions for laser powder bed fused IN718. It was found that the as-printed IN718 is characterized by very fine columnar/cellular dendrites with Laves phase precipitating at the grain boundaries as well as inter-dendritic regions, which differs from the microstructure of wrought and cast materials and requires special heat-treatment conditions different from the standard treatments. The results reveal that the relatively short homogenization treatment at 1080 °C for 1 h was not enough to significantly change the as-printed grain structure and completely dissolve the segregates and Laves phase. However, a completely recrystallized IN718 material and more Laves phase dissolution were obtained after homogenization treatment for 4 h. A further increase in time of the homogenization treatment (7 h) resulted in grain growth and coarsening of carbides precipitates. The solution treatment time at 980 °C did not cause noticeable changes in the crystallographic texture and grain structure. Nevertheless, the amount of δ-phase precipitation was significantly affected by the solution treatment time. After applying the heat-treatment time window, the hardness increased by 51–72% of the as-printed condition depending on the treatment time due to the formation of γ′ and γ″ in the γ-matrix. The highest material hardness was obtained after 1 h homogenization, whereas the prolonged time treatments reduced the hardness. This study provides a comprehensive investigation of the post heat-treatments of the laser powder bed fused IN718 that can result in an optimized microstructure and mechanical behavior for particular applications.

## 1. Introduction

Inconel 718 is a nickel-based superalloy which was developed by the International Nickel Company in 1959 [1]. Over the past decades, IN718 superalloy experienced outspread evolution in different industrial applications such as aerospace and energy industries [2]. This is due to its outstanding properties such as high strength, high resistance to corrosion, wear and oxidation at both low and elevated temperatures up to 650 °C [3,4]. Hence, IN718 has been implemented in several high-temperature applications such as the production of a majority (50 wt.%) of turbine engine components: rotor disc and turbine blades [5]. Solid solution strengthening and precipitation hardening are the principle strengthening mechanisms for IN718 superalloys [6]. IN718 is a multiphase superalloy that consists of primary face-centered-cubic (FCC) γ-phase matrix along with other secondary phases such as FCC γ′ (Ni_3_(Al,Ti)), body-centered-tetragonal (BCT) γ″ (Ni_3_Nb), orthorhombic δ-phase (Ni_3_Nb), hexagonal Laves phase (Ni,Fe,Cr)_2_(Nb,Mo,Ti) and (MC, M_23_C_6_, M_6_C) carbides [7]. Both γ′ and γ″ are the primary strengthening phases of the γ-matrix, whereas δ and Laves phases are known to degrade the mechanical properties in this alloy [7]. It is important to note, however, that the presence of an appropriate amount of δ-phase along the grain boundaries prevents undesired grain growth and improves the strength of the grain boundaries [2]. The microstructure and mechanical properties of IN718 superalloy are sensitive to the types, contents, distribution, and size of these precipitates [8]. Therefore, studying post-printing heat-treatments thoroughly is very important.

To date, IN718 components are usually fabricated using conventional manufacturing methods. However, several challenges face the manufacturing industries, such as excessive tool wear, high material waste, high production cost and long manufacturing time, because of the high hardness and poor machinability of IN718 superalloy [3,9]. Moreover, the extreme complexity of most of the IN718 components, such as turbine blades with internal cooling channels, requires long processing time and high manufacturing cost using the conventional techniques. Also, some of the existing manufacturing techniques, such as casting, require high temperature during the processing of IN718 parts which consequently results in macrosegregation of the Nb and Mo as reported by Hosseini et al. [10]. These challenges have become the motivation to look for new, more effective, manufacturing techniques for the IN718 superalloys. Additive manufacturing is one of the proposed and promising techniques to fabricate IN718 parts.

The laser powder bed fusion (LPBF) process is one of the additive manufacturing (AM) techniques that utilizes a layer-by-layer fabrication method to produce three dimensional (3D) metallic components using a high-intensity laser beam [5]. LPBF has attracted significant attention in the processing of advanced engineering materials such as aluminide, titanium alloys and nickel-based alloys [3]. This is due to its high potential to fabricate complex shape components without the need for specific tooling. Furthermore, the LPBF process offers several advantages compared to conventional subtractive manufacturing methods such as a reduction in manufacturing time, lower buy-to-fly ratio and lower production cost [11,12,13]. Moreover, dense metallic components, with a high relative density, up to 99.7%, can be achieved using LPBF at the optimal laser process parameters [14]. Thus, the LPBF process is fast becoming an attractive process for the fabrication of high-valued and critical IN718 components in various industries. Despite the great promises that the LPBF process has clearly exhibited, some inherent manufacturing drawbacks remain, such as high-level of residual stresses, elemental segregation, microstructure heterogeneity and anisotropic mechanical properties that should be overcome to fully realize and maximize the LPBF process’s potential [3,15,16,17,18]. To achieve the required microstructure and mechanical properties, thermal post-processing is required to obtain homogenous microstructure and defect-free components [7].

Numerous studies have been conducted to investigate the effect of thermal post-processing treatments on the microstructure and mechanical properties of 3D printed IN718 parts. Nevertheless, producing laser powder bed fused IN718 components with acceptable mechanical properties and cracking resistance considering the harsh in-service conditions has not been achieved yet [2,19,20]. According to the Aerospace Material Specification (AMS) of IN718, there are two industrial standard heat-treatments, AMS 5662 for wrought IN718 and AMS 5383 for cast IN718 [21,22]. The AMS 5662 treatment cycle includes solution anneal (980 °C for 1 h, air cooling) followed by aging treatment (720 °C for 8 h, furnace cooling at 55 °C/h to 620 °C, 8 h, air cooling) which is designated as SA [20,22], whereas AMS 5383 treatment includes homogenization treatment (1080 °C for 1.5 h, air cooling) followed by solution treatment (980 °C for 1 h, air cooling) + aging treatment (720 °C for 8 h, furnace cooling at 55 °C/h to 620 °C for 8 h, air cooling) which is designated as HSA [20,21]. Through literature, the majority of conducted researches on the thermal post-processing of laser powder bed fused IN718 were focusing on studying the effects of these standard treatments on the microstructure and mechanical properties. However, the microstructure, precipitates and texture of IN718 printed by LPDF are considerably different from those of the conventionally manufactured by forging and casting. This is because of the rapid solidification process associated with the LPBF process [7]. Therefore, these standard heat-treatments are expected to require some changes to become suitable for IN718 components fabricated by LPBF. The present work is aimed at helping to achieve this objective.

Zhang et al. [20] investigated the effect of the standard SA treatment of the wrought IN718 (AMS 5662) on the microstructure and mechanical behavior of the laser powder bed fused IN718 alloy. Their results show that the strength of as-printed specimens was lower than that of the wrought IN718 [20]. After SA treatment, the strength became comparable to that of the wrought alloy, but the ductility dramatically decreased in contrast with the ductility of wrought IN718. They attributed this behavior to a larger amount of δ-phase precipitates inside the grains and at the grain boundaries [20]. It was observed that the time scale of the δ-phase precipitation in nickel-based superalloy fabricated through LPBF is much shorter than in the wrought alloy (minutes versus tens to hundreds of hours) as reported by Fan et al. [17]. The faster precipitation of δ-phase in the laser powder bed fused IN718 was attributed to the non-uniform distribution of Nb in the as-printed state, since δ-phase usually precipitates in Nb-enriched areas [7,17,20]. Thus, the standard treatments of wrought IN718 alloy should not be expected to result in the optimum conditions and improve the microstructure and mechanical properties of IN718 processed by LPDF.

Furthermore, Deng et al. [2] studied the effect of a standard HSA treatment for cast IN718 (AMS 5383) on the microstructure and mechanical properties of IN718 samples fabricated by LPBF. Their results show that the HSA-treated microstructure still had brittle intermetallic Laves phase which resulted in the formation of microvoids during tensile testing and consequently deteriorated the mechanical behavior [2]. In addition, larger size and volume fraction of the δ-phase precipitated after the HSA conditions which resulted in lower ductility. The authors [2] concluded that homogenization treatment at 1080 °C for 1 h was not enough to completely dissolve the Nb and Ti-rich segregates and Laves phase. Also, they mentioned that the 1 h holding time of the solution treatment at 980 °C resulted in the precipitation of a larger amount and coarser size of δ-phase, which consequently consumed more Nb at the expense of precipitation of the strengthening γ″ phase [2]. However, some beneficial effects of the presence of δ-phase particles at the grain boundaries can be noted, including dislocation pinning and grain growth inhibition, when the amount and size of δ-phase particles are controlled [2,19]. In this context, Deng [1] reported that the presence of approximately 4% of δ-phase at the grain boundaries could effectively inhibit the grain growth during the in-service conditions. Therefore, it is desirable that the sought after heat-treatments result in grain boundary δ-phase and avoid its precipitation inside the grains.

Similarly, Dongyun et al. [19] applied the standard HSA and HA (1080 °C for 1.5 h, air cooling + 720 °C for 8 h, furnace cooling at 55 °C/h to 620 °C for 8 h, air cooling) heat-treatments to LPBF-processed IN718 alloy in order to analyze the effect of δ-phase on the mechanical behavior. Their results reveal that after the HSA treatment, the dendritic microstructure transformed to recrystallized grains in addition to precipitating fine δ-phase and MC carbides along the grain boundaries [19]. Also, they confirmed that δ-phase did not form in the matrix after the HA treatment [19]. Thus, the HA-treated samples exhibited improved mechanical behavior as compared to the HSA-treated samples [19]. The authors [19] attributed this trend to the fact that the Nb is the principle element necessary for the formation of both γ″ strengthening phase and δ-phase. Therefore, at a certain concentration of Nb in the matrix, δ-phase is formed at the expense of γ″-phase during the HSA heat-treatment, thus reducing the mechanical properties [19].

On the basis of the aforementioned findings, applying the industrial standard heat-treatment conditions of the cast IN718 superalloy (ASM5383) has some beneficial effects on the microstructure and mechanical behavior of the as-printed IN718. However, the times of the homogenization and solution treatments still need to be optimized further to obtain more homogeneous microstructure and improve the combination of strength and ductility in laser powder bed fused IN718 parts. Therefore, in the present study, a window of heat-treatment time has been established to optimize the post-treatment conditions for the printed IN718 by homogenizing the microstructure, dissolving the segregated elements and the brittle precipitates, and to improve the mechanical behavior.

This window covers a range of soaking times of both homogenization and solution treatments to investigate their effects on the microstructure, phase formation/dissolution, texture evolution and mechanical behavior of the 3D printed IN718. The soaking time of the homogenization treatment at 1080 °C was varied from 1 to 7 h, whereas for the solution heat-treatment at 980 °C, it was from 15 to 60 min. In addition, a mid-point condition, at 4 h of the homogenization and 37.5 min of the solution heat-treatment times, was selected to follow the microstructure and mechanical properties changes within this window. This approach could help to achieve the industrial standard conditions of the laser powder bed fused IN718 that have not been reported in the literature. It also provides a thermal post-processing map to control the precipitation of Laves, δ-phase and carbides which is essential for optimizing the printed IN718 mechanical behavior. The microstructure, compositional analysis, texture analysis and phase identification are studied using optical microscopy (OM), scanning electron microscopy (SEM), energy-dispersive spectroscopy (EDS), electron backscatter diffraction (EBSD) and X-ray diffraction (XRD) techniques. As for the mechanical behavior evaluation, Vickers microhardness measurements of the as-printed and post-treated specimens were carried out.

## 2. Experimental Procedure and Materials

### 2.1. Material and Manufacturing Methods

**Figure 1:**
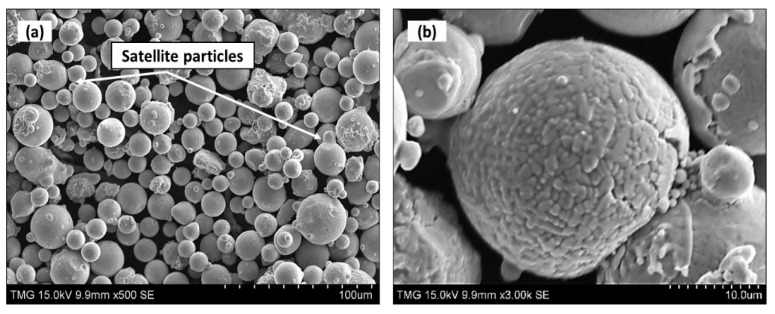
(**a**,**b**) Secondary electron scanning electron microscopy (SEM) micrograph of Inconel 718 (IN718) powder.

Gas-atomized IN718 powder, supplied by EOS-GmbH (Krailling, Germany), with a particle size distribution of D10 (18.2 μm), D50 (32.4 μm) and D90 (54.2 μm) was used as raw material to fabricate IN718 test samples via the LPBF process. The nominal chemical composition of the as-received powder is given in Table 1 and the SEM micrographs of the as-received powder are shown in Figure 1. As can be seen in Figure 1a, IN718 powder particles are dominantly spherical and some particles have a deviation from the spherical shape that is caused by smaller satellite particles. In the high-magnification SEM image in Figure 1b, a dendritic microstructure is observed on the surface of the particles which could be attributed to the rapid solidification during the gas atomization process [23].

**Figure 2:**
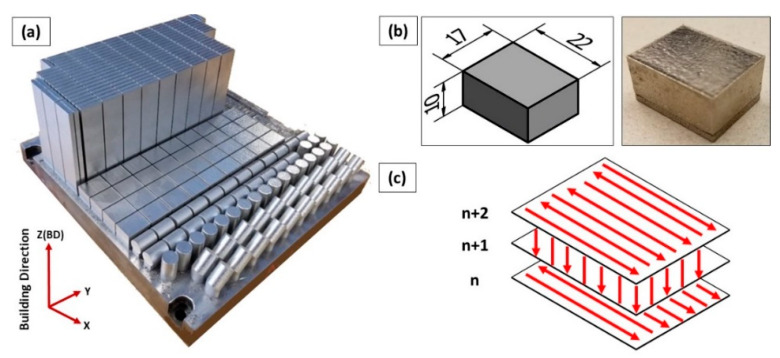
(**a**) LPBF building platform of IN718 displaying the building orientation; (**b**) schematic drawing and real as-printed IN718 test samples after WEDM removal; (**c**) schematic illustration of the laser scanning strategy in this study.

An EOS M280 (EOS, Krailling, Germany) LPBF system equipped with Yb:YAG 400 W fiber laser system was utilized to fabricate IN718 test samples using the EOS Original Parameter Set IN718_Surface 1.0 (285 W laser with 100 µm beam diameter, 1000 mm/s scanning speed, 110 µm hatching space with 67° hatching angle, and 40 µm layer thickness). The building platform was pre-heated to 80 °C to reduce the thermal gradient along the building direction, thus reducing thermal stresses in the produced parts. For this research, a set of cuboid specimens with a size of 22 × 17 × 10 mm^3^ were printed to investigate the microstructure evolution and for phase identification, as shown in Figure 2a,b. Figure 2c shows the laser beam scanning strategy used in this study. A controlled argon atmosphere was used to minimize the possibility of metal oxidation during the printing process. After the printing, all samples were removed from the building platform using wire electro-discharge machining (WEDM) in the as-built conditions without stress relief.

### 2.2. Thermal Post-Treatments

Thermal post-processing was applied to the as-printed IN718 samples to homogenize the microstructure and control the dissolution and precipitation of IN718 phases. To this end, a thermal post-treatment time window has been established to obtain the suitable treatment conditions for the laser powder bed fused IN718 superalloy. This post-treatment time window combines a wide range of soaking times of both homogenization and solution heat-treatments. The ranges of the homogenization and solution treatments soaking times were selected based on the transformation-time–temperature (TTT) diagram of the IN718 welds [23] and after studying the several heat-treatment studies in the literature.

**Figure 3:**
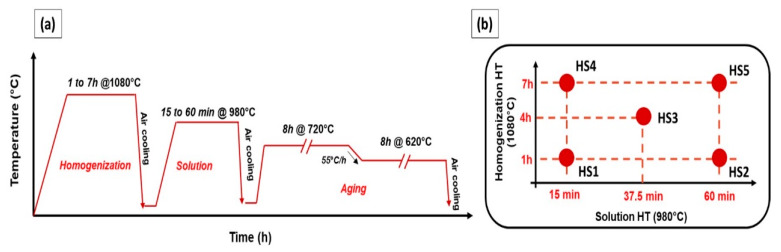
(**a**) Schematic representation of the entire heat-treatment cycle of the laser powder bed fused IN718 in the present study; (**b**) schematic drawing illustrates the position of the homogenization and solution treatment conditions inside the post-treatment time window.

Figure 3a,b shows the heat-treatment cycle and the window of the treatment time, respectively. As can be seen in this figure, the homogenization heat-treatment was performed at 1080 °C with soaking times of 1, 4 and 7 h, followed by air cooling in order to effectively homogenize the segregated elements and dissolve Laves phase. Then, the solution heat-treatment was carried out at 980 °C with soaking times of 15, 37.5 and 60 min, followed by air cooling, to control the size and amount of δ-phase precipitates. So, a total of five treatment conditions have been applied to the as-printed LPBF IN718 as listed in Table 2. The details of the five treatment conditions are as follows: among the five treatment conditions, two samples were homogenized at 1080 °C for 1 h and one of them was followed by solution treatment at 980 °C for 15 min, while the other was followed by solution treatment for 60 min at the same temperature, HS1 and HS2, respectively. Similarly, two other samples were homogenized at 1080 °C for 7 h and one of them was followed by solution treatment at 980 °C for 15 min, while the other, was for 60 min, HS4 and HS5, respectively. In addition, a mid-point for both the homogenization and solution soaking times was selected at 4 h of homogenization and 37.5 min of solution treatment, HS3.

Then, the five post-treated samples were aged under the same conditions of 720 °C for 8 h, furnace cooled by 55 °C/h to 620 °C, then held for 8 h at 620 °C, followed by air cooling—in order to study the effect of the preceded homogenization and solution heat-treatments on the precipitation of the strengthening phases and the mechanical properties of laser powder bed fused IN718. All heat-treatment conditions were performed using an electric-resistance furnace using a set of K-type thermocouples for monitoring the temperature of the IN718 samples during the treatments where the temperature difference was controlled within ± 5 °C.

### 2.3. Characterization Methods

Microstructure examinations of the IN718 in the as-printed and heat-treated conditions were carried out using a MEIJI TECHNO optical microscope (OM, San Jose, CA, USA) equipped with a 3.0 MP camera and scanning electron microscope, SEM (HITACHI S-3400N, Minato, Tokyo, Japan) equipped with an energy dispersive X-ray spectrometer, EDS at 15 kV. The chemical analysis of all primary and secondary phases was carried out using semi-quantitative EDS. Phase analysis of the as-printed and heat-treated samples was performed using XRD, (PANAnalytical X’pert Pro X-ray diffractometer, Lelyweg 1, Almelo, The Netherlands) with a CuKα radiation at 45 kV and 35 mA. The scanning range (2θ) from 30° to 100° was selected to include the maximum number of possible diffraction peaks. It is worth mentioning that all the XRD analysis was performed on the xy-plane (perpendicular to the building direction) of the as-printed and heat-treated samples.

For the metallographic analysis, the as-printed and heat-treated samples were sectioned in both xy-plane and xz-plane (parallel to the building direction) using a slow cutter (Buehler, Lake Bluff, IL, USA) with a mineral oil bath to prevent heat generation. The cut samples were mounted using conductive hot epoxy resin which then were mechanically ground gradually from 320 to 1200 grit using SiC abrasive papers and polished down to 0.5 µm using alcohol-based diamond suspension. To reveal the precipitated phases and dendritic microstructure, the polished samples were etched using a solution of 10 mL hydrochloric acid + 1.5 mL 30% hydrogen peroxide [2]. Electron backscatter diffraction analysis (EBSD, SU-8230 Hitachi SEM equipped with Bruker e-Flash HR EBSD detector, Minato, Tokyo, Japan) was used to analyze the microstructure texture evolution, grain size and grain shape distributions in as-printed and after different post-treatment conditions. For the EBSD analysis, the specimens were polished manually down to 0.5 µm grit size, then a vibromet polishing with a 0.05 grit size colloidal silica for 24 h. An IM4000Plus Hitachi ion milling was used to eliminate the residual scratches and deformed surfaces using process parameters: 6 kV accelerating voltage and 1.5 kV discharge voltage for 40 min. For consistency, all EBSD maps were acquired at the center of the cuboid IN718 samples. In the EBSD analysis, the IN718 test samples were scanned at 20 kV and the pixel size of 1.62 μm. It is worth mentioning that the total map size for each condition was 1298 × 973.4 μm^2^ surface area to cover the maximum number of grains (>1500).

### 2.4. Room Temperature Mechanical Testing

Room temperature mechanical properties of the as-printed and heat-treated conditions were evaluated using the Mitutoyo, MNK-H1 Vickers microhardness tester (Aurora, IL, USA) under a load of 500 gf and a dwell time of 15 s. All tested samples were ground and polished as required for the microhardness test [2]. Microhardness testing was conducted in two stages of before and after aging treatment. At least 15 evenly distributed measurements for each condition were performed to obtain accurate results. All indentations were performed on the xy-plane of the cuboid samples.

## 3. Results and Discussion

### 3.1. Microstructure of the As-Printed Condition

**Figure 4:**
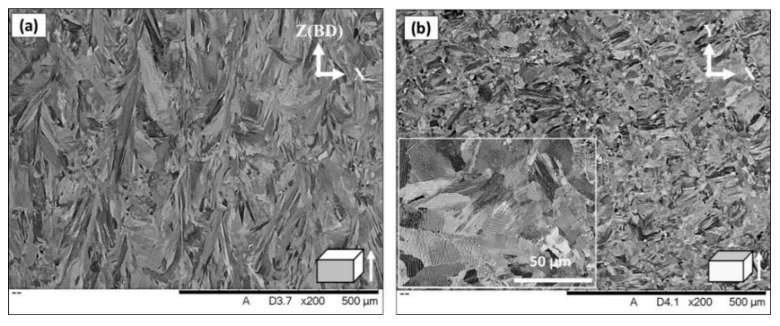
SEM micrograph of the non-etched as-printed laser powder bed fused IN718 superalloy: (**a**) vertical plane (xz); (**b**) horizontal plane (xy).

The microstructure of the vertical (xz) and horizontal (xy) planes in the as-printed condition were analyzed as shown in Figure 4a,b, respectively. The examined surface, with respect to the building direction, was indicated in the lower right corner of each SEM micrograph. As can be seen on the xz-plane in Figure 4a, the morphologies of the grains appear relatively elongated and the longitudinal axes of these grains are approximately parallel to the building direction. This microstructure is attributed to the high dependence of the grain size and morphologies on the intensity and direction of the thermal gradient during the printing process. The temperature difference between the upper level of the powder bed, where the local laser heating is at its maximum, and the lower level which is close to the building platform results in high heat dissipation and thermal gradient along the building direction. Besides, the thermal conductivity of the dense material (previously solidified layers) is roughly 100 times higher than that of the powder material (adjacent to laser track) which results in higher heat dissipation in the -z direction [19]. Thus, during the solidification stage, the grains grow along the heat dissipation direction and form such a columnar grain structure. By contrast, in the xy-plane, equiaxed grains morphology are observed which corresponds to the transverse cross-section of the elongated grain structure, as shown in Figure 4b. Moreover, very fine precipitates, a few hundred nm in size, embedded inside the grains and along the grain boundaries are seen in the magnified inset of Figure 4b.

**Figure 5:**
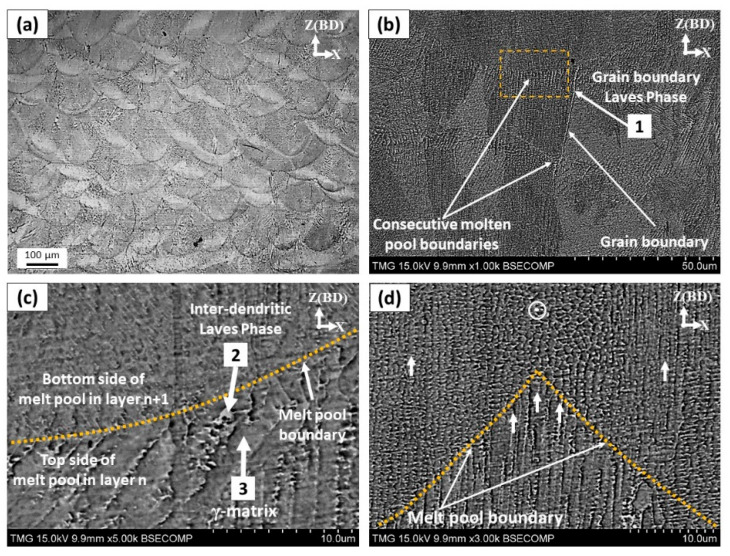
(**a**) Optical microscope (OM) micrograph of the vertical plane with arc-shape melt pool boundaries; SEM micrograph of the etched as-printed IN718 in the vertical plane showing: (**b**) columnar grains with epitaxial growth across several layers; (**c**) dendrite size at the melt pool boundaries; (**d**) changes in the dendrite direction in the overlapping zone. Arrows 1, 2 and 3 indicate the EDS spot analysis of grain boundaries Laves phase, inter-dendritic Laves and γ-matrix, respectively, as listed in Table 3.

**Figure 6:**
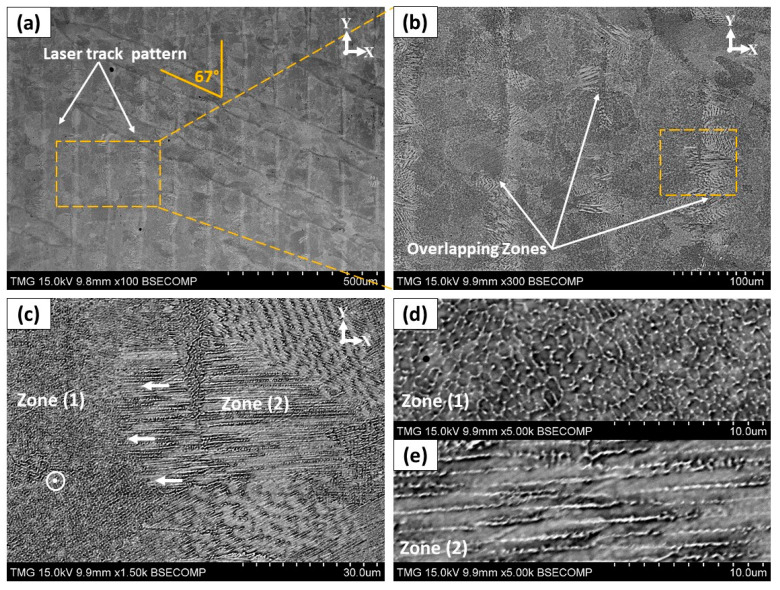
SEM micrograph of the etched as-printed IN718 in the horizontal plane showing: (**a**) the laser beam track and scanning strategy; (**b**) high magnification view for the area marked in (**a**); (**c–e**) changes in the dendrite directions in the horizontal overlapping zone as indicated in (**b**).

To characterize the dendritic microstructure and obtain further details about the as-printed substructure, both the xz and xy-planes were etched and examined as shown in Figure 5 and Figure 6, respectively. After etching, the melt pool boundaries can be clearly seen in the OM micrograph of the xz-plane in Figure 5a. The shape of the molten pool appears as arc-shaped contours facing upwards along the building direction, which reflects the Gaussian distribution shape of the laser beam intensity. This is the typical microstructure of the laser powder bed fused IN718 superalloy as also reported in [7,24,25]. The microstructure of each individual melt pool consists of very fine columnar dendrites as shown in Figure 5b–d. The growth direction and size of the dendrites in the molten pool depend on the direction of the local thermal flux and the cooling rate, respectively [26]. Moreover, the <100> texture is the preferred crystallographic orientation for the FCC structure [2]. This strong texture was observed to be preferential for many FCC engineering materials, built using the laser and electron beam powder bed fusion (PBF) processes [27,28]. Therefore, anisotropic mechanical properties are expected for the as-printed IN718 superalloy. Also, as shown in Figure 5b, some of these columnar dendrites extend across several melt pools, suggesting that the epitaxial growth of new dendrites occurred upon the preexisting dendrite colonies of the solidified grains along the building direction. This is because, during the laser beam scanning, the laser radiation causes extremely fast melting of the current powder layer and also partial melting of the previously solidified layer. After the laser beam leaves the melt pool, rapid cooling of the molten pool and rapid solidification occur with epitaxial growth. Such epitaxial growth crossing over several melt pool boundaries is widely reported in the literature [2,26,29]. This kind of growth indicates a strong bonding between the solidified layers due to the lower lattice mismatch that consequently reduces the risk of the inter-layer delamination [26]. However, such elongated grain morphology along the building direction results in strong anisotropic mechanical properties.

The microstructure inside the melt pool (Figure 5c) is inhomogeneous, as the fine dendritic microstructure is observed at the bottom of the melt pool that gradually changes to be coarser dendrites towards the top of the molten pool. This confirms the microstructure inhomogeneity that consequently leads to anisotropic mechanical properties as reported by Seede et al. [30]. The change in the size of the dendrites can be attributed to the change in the cooling rate across the melt pools during the solidification stage [26] and also the fact that some dendrites have been engulfed by those which are better aligned crystallographically opposite to the heat flux direction. This provides a wider area for each dendrite to grow and develop the arms [31]. Furthermore, at the beginning of the solidification process, the cooling rate of the bottom part of the melt pools, in contact with the solid substrate, is relatively high but gradually decreases towards the top side which promotes the constitutional undercooling respectively. Therefore, the bottom part experiences an earlier solidification leading to the formation of fine and narrow dendrites that slightly increases in size towards the top border. Such an inhomogeneous microstructure was also reported by Calandri et al. [26].

In the xy-plane shown in Figure 6a, traces of the laser beam passes are observed to have 67° between two adjacent layers that reflect the laser scanning strategy. The width of each laser track is found to be approximately near the diameter of the laser beam (≈100 µm). As shown in Figure 6b–e, the microstructure of the overlapping zone is different from that in the center of the laser track. In the center of the laser tracks (zone 1), the growth direction of the cellular dendrites does not change as it stays oriented along the building direction as shown in Figure 6d. But at the laser track edges (zone 2), dendrites tend to even up to rotate 90° with respect to the building direction as shown in Figure 6e. Such a microstructure changes between the center and the edge of the laser tracks is also observed in the xz-plane, as shown in Figure 5d. This is because of the local variations in the direction of the thermal gradient and heat flux towards the center of the melt pool. At the boundaries of the laser tracks, the thermal flux towards the center of the molten pool is greater than along the building direction due to the Gaussian shape of the laser beam intensity. Such a change in the dendrites growth direction at the laser tracks boundaries has also been reported by Mostafa et al. [22] and Calandri et al. [26].

**Figure 7:**
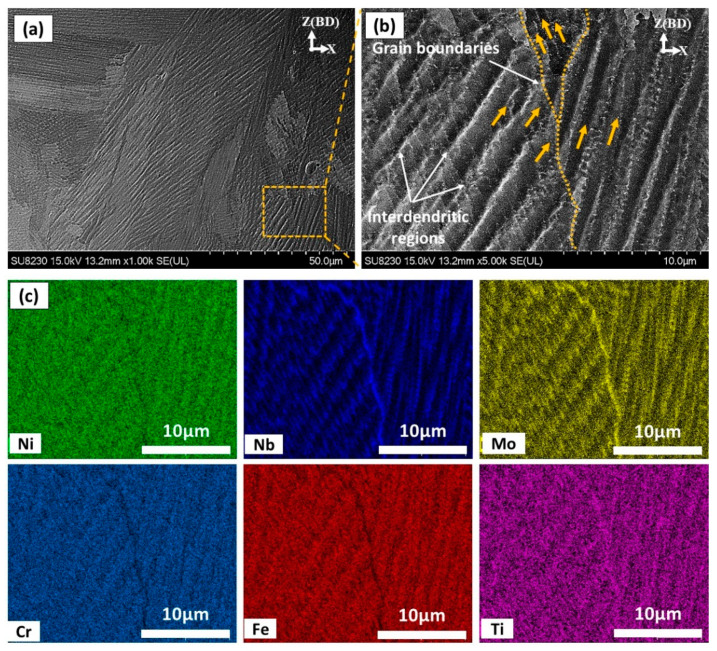
(**a**,**b**) SEM micrograph of the as-printed condition illustrating the segregates in the inter-dendritic and grain boundary regions; (**c**) EDS elemental distribution in the areas indicated in (**b**).

Figure 7 illustrates the elemental mapping of the as-printed IN718 using the EDS analysis to determine the chemical composition of the inter-dendritic (white phase) and the dendritic (dark phase) zones. As can be seen in Figure 7c, high concentrations of Nb, Ti and Mo are observed in the inter-dendritic regions in comparison with the primary dendrites along with depletion in Ni, Fe and Cr. The concentration of Nb, Ti and Mo at these precipitates reached up to 5.4, 3.2 and 1.1 times, respectively, higher than that of the dendrites, but they are depleted in Ni, Fe and Cr with respect to the nominal composition of the matrix as can be inferred from Table 3. This is attributed to the solute rejection and element redistributions during the solidification process. During the solidification process of IN718, segregation of Nb in the inter-dendritic region occurs due to its partitioning behavior, and the solidification sequence is well established as follows: Liquid → γ → γ + NbC → γ + NbC + Laves [2]. However, the amount and size of these Nb-precipitates in the laser powder bed fused IN718 superalloy are much lower and smaller than those in the cast IN718 as reported by Zhang et al. [19]. This is because the amount and the size of precipitates are significantly minimized by increasing the cooling rate [2,19]. Such microsegregation of Nb, Ti and Mo results in the formation of brittle intermetallic Laves phase along the grain boundaries and in the inter-dendritic regions. According to the work of Tucho et al. [7], Laves phase formation requires, approximately, 10 wt.% Nb. Therefore, the formation of brittle intermetallic Laves phases consumes most of the segregated Nb and therefore depletes it from the matrix, which is confirmed by the current EDS analysis. The presence of such large amounts of Laves phases in the inter-dendritic regions and at the grain boundaries is detrimental to the mechanical properties. Thus, further heat-treatments are required to dissolve these precipitates and microsegregates. Besides, the precipitation of the strengthening phases (γ′ and γ″) and δ-phase is not observed in the as-printed microstructure, which is in agreement with the results obtained by Gao et al. [5]. This is attributed to the extremely high cooling rates of the LPBF process.

**Figure 8:**
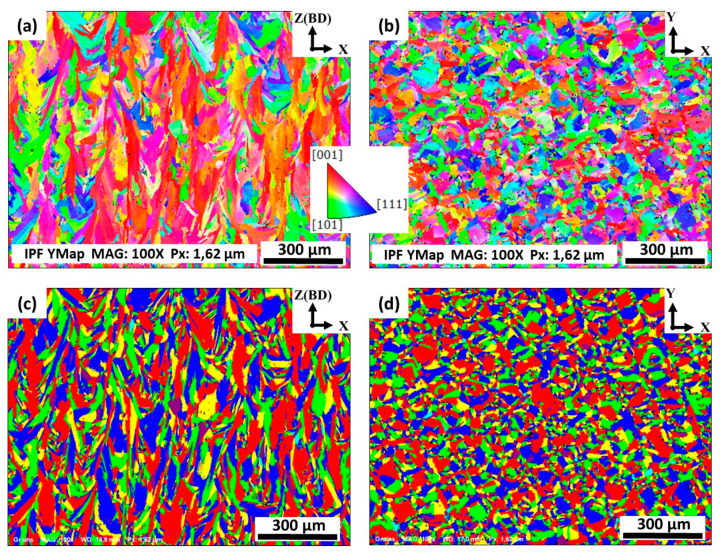
Inverse pole figures (IPF) (**a**,**b**) and grain maps (**c**,**d**) of the as-printed laser powder bed fused IN718: (**a**,**c**) xz-plane; (**b**,**d**) xy-plane. The EBSD mappings of the as-printed samples were plotted using the y-based projected IPF.

To assess the material textures, grain orientations and morphologies of the laser powder bed fused IN718 in the as-printed condition, EBSD mappings were acquired from both the xz and xy-planes. Also, inverse pole figure (IPF) and grain maps were constructed from the EBSD data to illustrate the microstructural anisotropy of both the xy and xz-planes, in the as-printed condition, as shown in Figure 8. For simplicity, the examined surface, relative to the building direction, is indicated in the upper right corner of each EBSD map in Figure 8. From the IPF in the xz-plane (Figure 8a), the as-printed LPBF IN718 exhibits strong texture (red area) along <100>, whereas in the xy-plane (Figure 8b), a non-distinct/weak texture is observed. Also, it can be confirmed that the microstructure of the as-printed condition in the xz-plane contains mainly elongated grains oriented parallel to the building direction as shown in Figure 8c. By contrast, the xy-plane mapping reveals a pattern of chessboard-like shape which can be related to the laser beam scanning strategy as shown in Figure 8d. Such as-printed features and grain morphologies are consistent with those obtained by Deng et al. [2].

### 3.2. Microstructural Evolutions with Heat-Treatment Time

**Figure 9:**
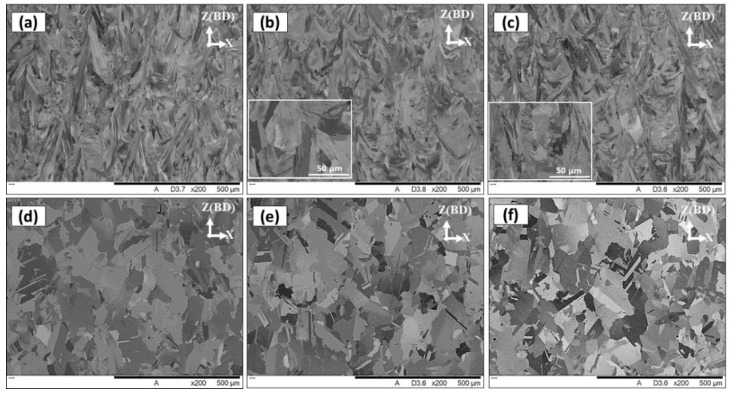
SEM micrograph of non-etched laser powder bed fused IN718 in conditions: (**a**) as-printed; (**b**) HS1; (**c**) HS2; (**d**) HS3; (**e**) HS4; (**f**) HS5.

As illustrated in Figure 5 and Figure 6, the as-printed microstructure contains elongated grains with large amounts of segregations and Laves phase at the grain boundaries and inter-dendritic regions which is detrimental to the mechanical behavior of the IN718 alloy. Applying the post-treatment time window in this study aims to optimize the homogenization and solution treatment time for laser powder bed fused IN718. Figure 9 shows the grain structure evolution after applying the five heat-treatment conditions with variations in the homogenization and solution treatments times. The as-printed micrograph is included in this figure for comparison. All microstructure analyses of the heat-treated conditions were carried out on the xz-plane before the aging step for better comparison. All heat-treatments before the aging step are designated by HS referring to the homogenization treatment (H) followed by the solution treatment (S). As can be seen in Figure 9b,c, after the homogenization treatment at 1080 °C for 1 h in both HS1 and HS2 conditions, the grain morphology did not significantly change from that in the as-printed condition and the microstructure still consisted of elongated grains oriented parallel to the building direction.

**Figure 10:**
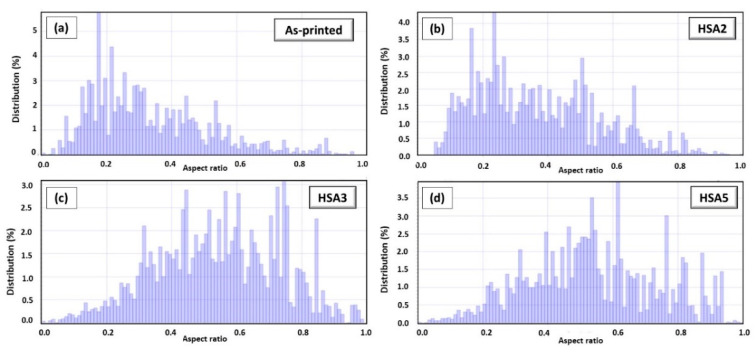
Grain morphology distribution displaying the change in grains aspect ratio as a function of heat-treatment conditions: (**a**) as-printed; (**b**) HSA2; (**c**) HSA3; (**d**) HSA5.

The EBSD-based grain structure aspect ratio measurements were performed in the as-printed and heat-treated conditions as shown in Figure 10. As seen in Figure 10a,b, the aspect ratio measurements demonstrate that the relatively short (1h) homogenization time retains the elongated grain structure of the as-printed specimens, which is consistent with SEM and EBSD results. This finding indicates that the heat input during the 1h homogenization treatment at 1080 °C is not sufficient for complete recrystallization which is driven by the residual stresses induced during the LPBF of IN718. It is worth mentioning that the recrystallization temperature of the (30–50%) cold-worked Inconel 718 is above 885 °C [32], while the results obtained in the present study reveal a significantly higher recrystallization temperature. This suggests that the level of induced residual stresses during the printing process is lower than that in the cold-worked condition in [32].

Figure 9c shows an illustrative microstructure of the sample after 4 h homogenization at 1080 °C in HS3. More equiaxed grains with an average grain size of 66 μm are observed in comparison with the as-printed and 1 h homogenized conditions suggesting that this treatment promoted a near complete static recrystallization. Moreover, a relatively higher aspect ratio was measured as compared with the 1 h homogenization time as shown in Figure 10c. This further confirms that the 4 h homogenization treatment at 1080 °C, in HS3, provides sufficient activation energy to break the intermetallic bonds and improve the diffusion which finally results in noticeable recrystallization in relation to other conditions with shorter holding times. The transformation of grain structure from columnar to equiaxed grains is necessary to achieve isotropic mechanical properties.

For the prolonged homogenization treatment (7 h), equiaxed grain structure with an average size of 75 μm were obtained, as shown in Figure 9e,f. This finding indicates that the recrystallization process has been completed and followed by a grain growth which is consistent with the aspect ratio analysis in Figure 10d. The comparison between the grains structure in both HS1 and HS2 conditions in Figure 9b,c reveals that the solution time at 980 °C did not significantly affect the grain morphologies. The same behavior is observed in the HS4 and HS5 conditions, as shown in Figure 9e,f.

**Figure 11:**
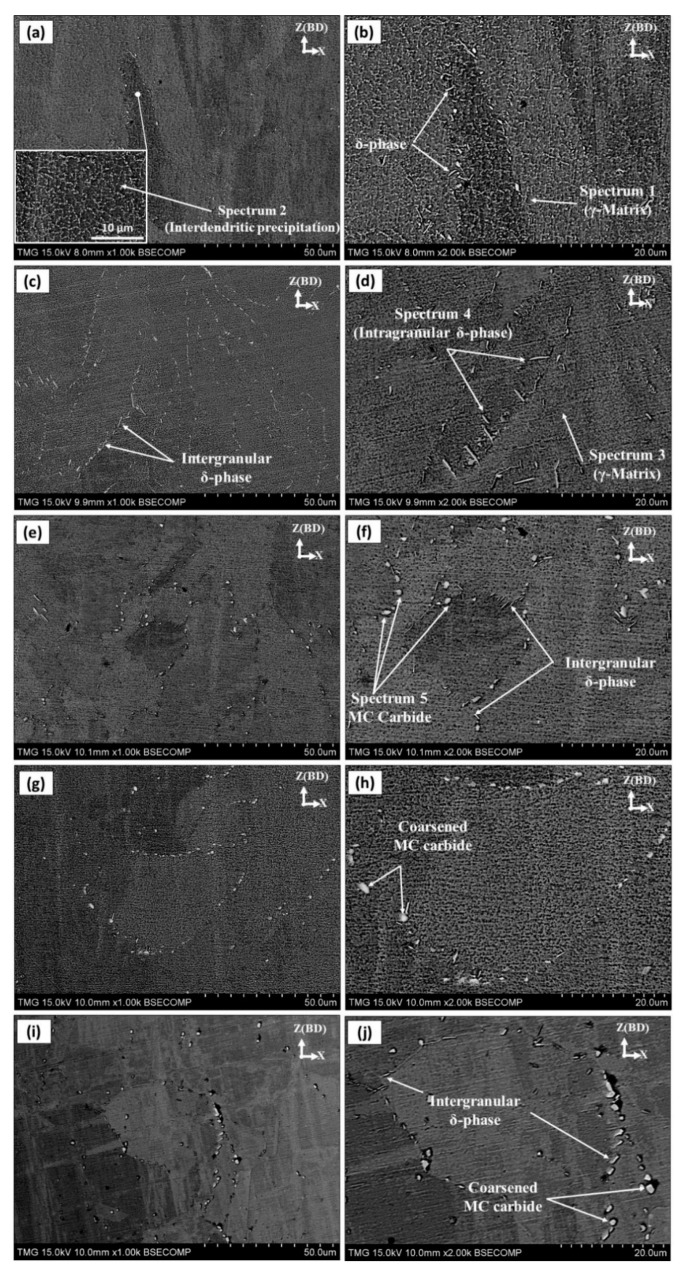
Microstructure of etched LPBF IN718 samples on vertical (xz) plane in heat-treated conditions: (**a**,**b**) HS1; (**c**,**d**) HS2; (**e**,**f**) HS3; (**g**,**h**) HS4; (**i**,**j**) HS5 treatments.

Figure 11 shows the microstructure evolution, in terms of segregated elements and phases dissolution and/or precipitation, as a function of the soaking time of both homogenization and solution treatments. Generally, as can be seen in this figure, the arc-shaped contours present in the as-printed condition (Figure 5a), that corresponds to the melt pool boundaries, completely disappeared after applying any of the five heat-treatment conditions. For the HS1 condition, Laves phase was partially dissolved in comparison with the as-printed condition. However, the cellular-dendritic microstructure in condition HS1 is still observed as shown in Figure 11a,b. The EDS point analysis for these dendrites shows the occurrence of Nb and Ti enrichment and depletion in Ni, Fe and Cr at the edge of the dendrites, compared with the γ-matrix (spectrum 1 and 2 in Table 4). This could be attributed to the fact that the 1h homogenization time of the HS1 condition is not enough to completely dissolve the segregated elements and Laves phase. Furthermore, limited amounts of δ-phase precipitated during the solution treatment at 980 °C for 15 min after the HS1 condition.

In the HS2 condition, the network of the cellular-dendritic substructure completely disappeared as shown in Figure 11c,d. Moreover, a large amount of needle-like δ-phase precipitated close to and along the grain boundaries as shown in Figure 11d. Since the homogenization treatment at 1080 °C for 1 h is not enough to completely dissolve the microsegregates of Nb and Ti in the inter-dendritic region (cellular substructure), intragranular nucleation and growth of the δ-phase are observed close to the grain boundaries. It is well known that the δ-phase preferentially precipitates at regions of Nb-enrichment [2]. This suggests that the concentration of the segregated Nb in the inter-dendritic region and along the grain boundaries was high enough to drive the formation of δ-phase through the subsequent solution heat-treatment process. This is in good agreement with Tucho et al. [7] who reported that δ-phase requires a localized concentration of at least 6–8 wt.% of Nb to precipitate. The absence of the cellular microstructure inside the grains, which were enriched by Nb, after HS2 in comparison with HS1 suggests that the segregated Nb is consumed to precipitate the intragranular needle-like δ-phase during the 1 h solution heat-treatment at 980 °C. The precipitation of the intragranular δ-phase has a detrimental impact on the mechanical behavior as reported by Trosch et al. [13]. Also, more precipitation of δ-phase consumes more Nb, which is an essential alloying element for the precipitation of γ″ strengthening phase. Therefore, the extent of the δ-phase precipitation has a direct impact on the mechanical behavior of the laser powder bed fused IN718.

EDS analysis was performed to identify the precipitated phases and follow the back diffusion of the segregated elements into the γ-matrix after HS1 and HS2 treatments. The average of at least five EDS spot analyses in the γ-matrix at different locations is reported in Table 4. As can be seen from Table 4, the concentrations of Nb, Ti and Mo in the γ-matrix of both HS1 and HS2 conditions increased in relation to the as-printed IN718 listed in Table 3. The relatively lower concentration of Nb in the HS2 with respect to the HS1 condition is attributed to the higher precipitation of δ-phase (Ni_3_Nb) after HS2.

The results reported in Figure 11e,f reveal that after the HS3 treatment, more Laves phase dissolution and complete dissolution of cellular-dendritic microstructure occurred in contrast to HS1. This indicates that with longer homogenization times (4 h), enough energy is provided to significantly dissolve elemental segregates and the Laves phase. However, more irregular-shaped precipitations along the grain boundaries are observed as shown in Figure 11e,f. According to the EDS analysis, these precipitates are highly enriched with Nb (≈52 wt.%), Ti (≈6 wt.%) and C and, therefore, indexed as carbides, as shown in Table 4. Although carbon content in these precipitates was found to be significantly higher than that in the matrix, specific values are not reported here due to the low accuracy of the EDS in measuring carbon, especially for such small precipitates. The presence of such coarse carbides along the grain boundaries has a detrimental effect on the mechanical behavior of the IN718 superalloy, especially at elevated temperatures. Furthermore, a moderate amount of needle-like δ-phase precipitated mainly along the grain boundaries as can be seen in Figure 11. By comparison, the amount and the size of the δ-phase were less than that precipitated in HS2 treatment. This again confirms that the amount and size of the δ-phase are directly proportional to solution treatment time. Also, it can be seen from Figure 11f that only intergranular δ-phase precipitated in the HS3 condition reflecting the fact that the homogenization at 1080 °C for 4 h completely dissolved the Nb-rich precipitates in the inter-dendritic regions [7].

Coarse irregular-shaped precipitates along the grain boundaries are observed after applying the prolonged (7 h) homogenization treatment at 1080 °C as shown in Figure 11g–j. According to the EDS analysis of these particles, enrichment in Nb, Ti and C is observed and identified as carbides according to Tucho et al. [7]. Despite that both the HS5 and HS2 treatments include the same solution time, 1 h, at 980 °C, the amount of δ-phase in the HS5 condition is less than that in the HS2 condition as shown in Figure 11c,i. This could be attributed to the long homogenization treatment in the HS5 condition that preceded the solution treatment. This significantly dissolved the segregated Nb along with Laves phase in the inter-dendritic regions and grain boundaries, resulting in a relatively uniform distribution of Nb in the matrix. Accordingly, the concentration of Nb at the grain boundaries after HS5 is not enough to precipitate the same amount of δ-phase after HS2 since the precipitation of δ-phase requires at least 6–8 wt.% of Nb as reported by Tucho et al. [7].

### 3.3. Structure, Texture and Phase Evolution

#### 3.3.1. Structure and Phase Evolution using XRD Analysis

**Figure 12:**
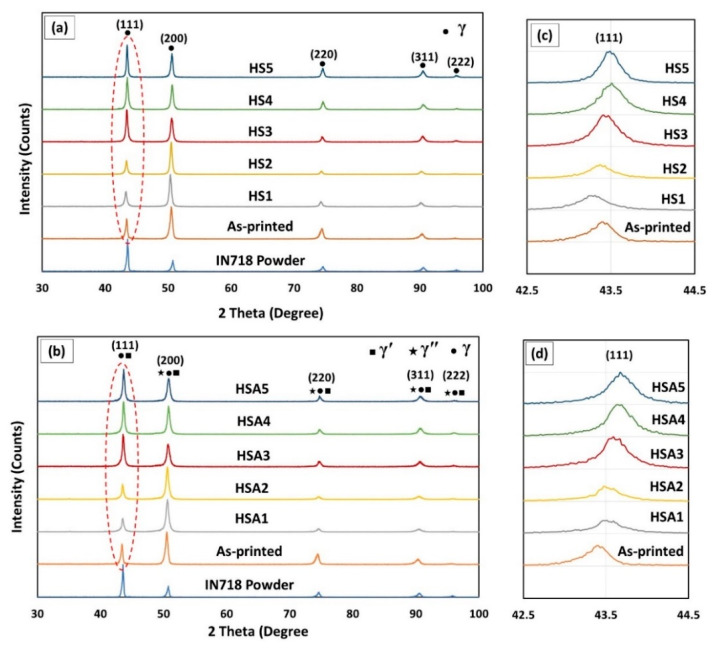
XRD patterns of the IN718 powder, as-printed LPBF and heat-treated conditions: (**a**) before aging; (**b**) after aging; (**c**,**d**) focus on 2θ = 43.3 in both (**a**,**b**).

The XRD patterns of the as-received IN718 powder, as-printed and heat-treated IN718 samples (before and after aging treatment) from the xy-plane are illustrated in Figure 12. The diffraction pattern of the as-received IN718 powder shows the main five peaks at diffraction angles of: 43.39° (111), 50.49° (200), 74.39° (220), 90.29° (311), 95.57° (222) that correspond to the γ-matrix [26]. Compared to the IN718 powder, the as-printed condition shows a higher peak intensity of (200) than (111) which is consistent with the commonly recognized texture for the laser powder bed fused IN718 superalloy. This is attributed to the preferred <001> crystallographic growth orientation of the γ (FCC) crystals and the presence of a large thermal gradient along the building direction [24].

Generally, after applying the five heat-treatments, changes in the peak intensities and peak positions are observed suggesting microstructural evolution in terms of texture and secondary phase dissolution and/or precipitation. Before aging treatment, the XRD patterns of the HS1 and HS2 conditions exhibited also higher peak intensity of γ (200) than of γ (111) suggesting that homogenization treatment for 1 h was not enough to change the as-printed strong texture along γ (200) as shown in Figure 12a. After the HS3, HS4 and HS5 treatments, the XRD patterns exhibited a different peaks intensity compared to the as-printed, HS1 and HS2 conditions. The intensity of the γ (111) peak became higher than that of the γ (200), indicating that significant change in the as-printed texture occurred after the HS3, HS4 and HS5 treatments. This suggests that homogenization treatment at 1080 °C for 4 h or more is sufficient to significantly change the as-printed texture. Figure 12b shows the diffraction pattern of the heat-treated conditions after aging. It can be observed that the XRD patterns of the post-processed conditions before and after aging appear identical in terms of texture, implying that the aging treatment did not cause noticeable changes in the material texture, which is in agreement with Wakshum et al. [33].

According to Bragg’s law, changes in the peak positions (2θ) are explained by variations in lattice spacing, which consequently refer to change in material composition [34]. Also, it is well recognized that the atomic size of Nb, Ti and Mo, which are the dominant segregated elements in the as-printed condition, are 33%, 18% and 27.5% larger than that of Ni, respectively [35]. This indicates that noticeable changes in lattice parameters are expected to occur during the dissolution and/or rejection of these solute elements. Thus, following the changes in the peak positions helps to follow the precipitation/dissolution of Nb, Ti and Mo during the heat-treatments [34]. Figure 12c,d shows the peak position of the γ (111) plane before and after aging treatment. Before aging treatment, the HS1 and HS2 conditions revealed a peak shift to smaller 2θ values in relation to the as-printed sample as shown in Figure 12c. This could be attributed to the dissolution (back diffusion) of the segregated alloying elements into the matrix during the 1h homogenization treatment. While after HS3, HS4 and HS5 treatments, peaks shifted to the higher 2θ values that could be explained by the depletion of the Nb, Ti and Mo in the γ-matrix. This behavior is consistent with the current microstructure and EDS results of the HS3, HS4 and HS5 treatments since after the application of these conditions, more precipitation of carbides is observed which consequently consumed more Nb and Ti from the γ-matrix. While after aging treatment, γ (111) of the γ-matrix in the five treatments conditions shifted to higher diffraction angles because of the precipitation of the strengthening phases, γ′ (Ni_3_ (Ti, Al)) and γ″ (Ni_3_Nb), that consumed Ti and Nb from the matrix.

**Figure 13:**
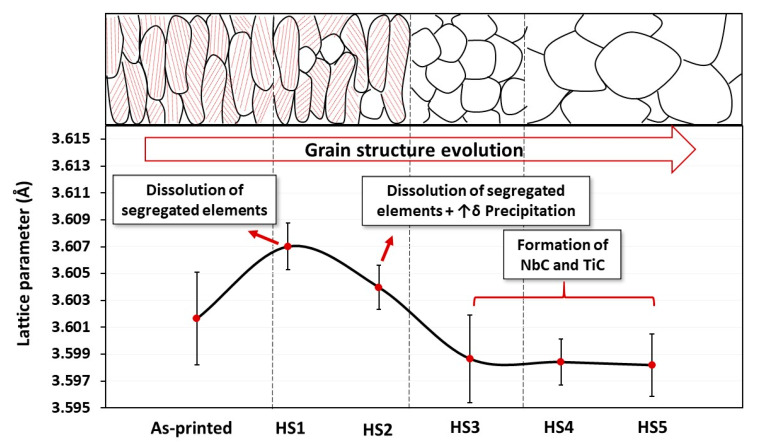
Evolution of the γ-matrix’s lattice parameter and grain structure as a function of the post-treatment conditions.

To analyze this further, the lattice parameter of the γ-matrix was calculated from the main five diffracted peaks in the as-printed and post-processed specimens before aging, and then the average value for each condition was calculated. Figure 13 shows the calculated lattice parameter of the γ-matrix and the grain structure evolution as a function of the heat-treatment conditions. After the HS1 and HS2 treatments, the lattice parameter of the γ-matrix increased from (3.5999 ± 0.0034 Å) in the as-printed condition to (3.6070 ± 0.0017 Å) and (3.6039 ± 0.0016 Å), respectively. This behavior is attributed to the back diffusion of the Nb and Ti into the γ-matrix during the 1h homogenization treatment in both HS1 and HS2 conditions that consequently resulted in an expansion of the lattice parameters. Among the HS1- and HS2-treated conditions, HS2 exhibited a relatively smaller lattice parameter. This is due to a larger amount of δ-precipitates (Ni_3_Nb) after HS2 treatment that depleted Nb from the γ-matrix. On the other hand, after the HS3, HS4 and HS5 treatments, reduction in the lattice parameter value with respect to the other conditions is observed and the lattice parameter stays relatively constant after these treatments due to the precipitation of Ti and Nb carbides, which is consistent with the microstructure and EDS analyses.

#### 3.3.2. Texture Evolution and Grain Structure Analysis

**Figure 14:**
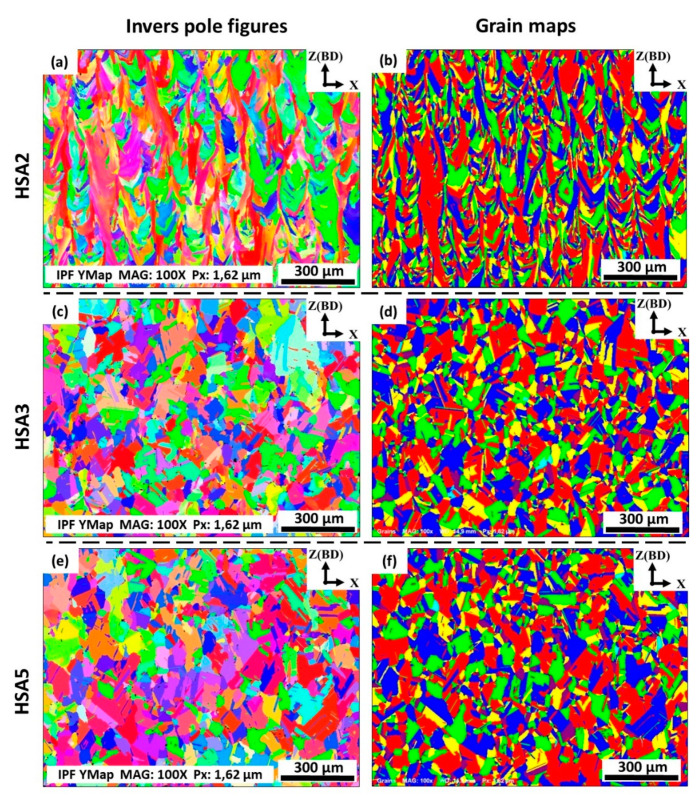
The EBSD inverse pole figures (**a**,**c**,**e**) and grain maps (**b**,**d**,**f**) in the xz-plane of laser powder bed fused IN718 specimens in conditions: (**a**,**b**) HSA2; (**c**,**d**) HSA3 and (**e**,**f**) HSA5. For the IPF color legend, please refer to that presented in Figure 8. All the EBSD crystallographic orientation maps were plotted using the y-based projected IPF.

Since producing materials with strong texture and anisotropic mechanical properties are considered to be one of the main drawbacks of the LPBF process of Inconel 718, crystallographic orientations of IN718 as a function of the homogenization and solution treatment times were examined aiming to obtain the treatment conditions that result in a weak material texture and isotropic mechanical properties. For that, EBSD analysis was carried out to evaluate the material textures, grain orientations and grain morphologies of the post-treated laser powder bed fused IN718 superalloy. According to the XRD results, it is found that the homogenization holding time significantly affects the crystallographic orientation of the as-printed IN718. Thus, the three levels of the homogenization time 1, 4 and 7 h, which corresponded to HSA2, HSA3 and HSA5 heat-treatments, were examined using EBSD mapping. All EBSD maps were acquired from the xz-plane, since the crystallographic orientation along the building direction is of main interest. It can be confirmed from Figure 14a that the HSA2 treatment in the present study did not significantly alter the crystallographic structure and orientations as the grains remained columnar and oriented parallel to the building direction which is consistent with the current microstructure and XRD results. This indicates that anisotropic mechanical properties are expected for the HSA2-treated samples.

On the other hand, after HSA3 and HSA5, a significant change in the crystallographic orientations and grain morphologies are observed as shown in Figure 14b,c. Non-distinct texture for laser powder bed fused IN718 components can be obtained using these conditions. The microstructure of the HSA3 and HSA5 conditions are characterized by the presence of mainly equiaxed randomly oriented grains with annealing twins, indicating that recrystallization has occurred as shown in Figure 14d,f, respectively. Such a change in grain morphologies and crystallographic orientations would have a beneficial effect on producing components with isotropic mechanical properties.

### 3.4. Room Temperature Vickers Hardness in Both the As-Printed and Heat-Treated Conditions

**Figure 15:**
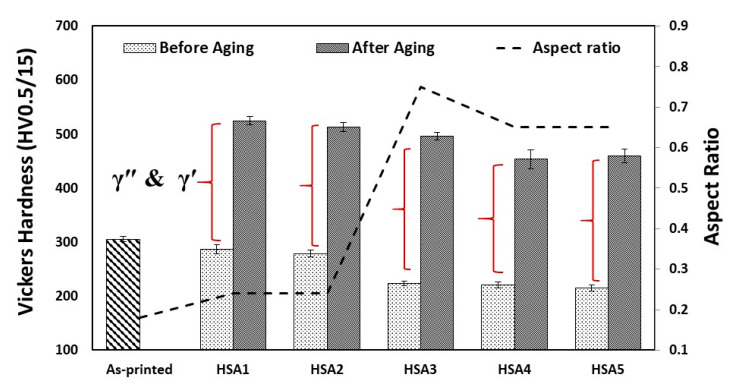
Vickers hardness and aspect ratio evolutions in the as-printed and post-processed samples before and after aging.

**Figure 16:**
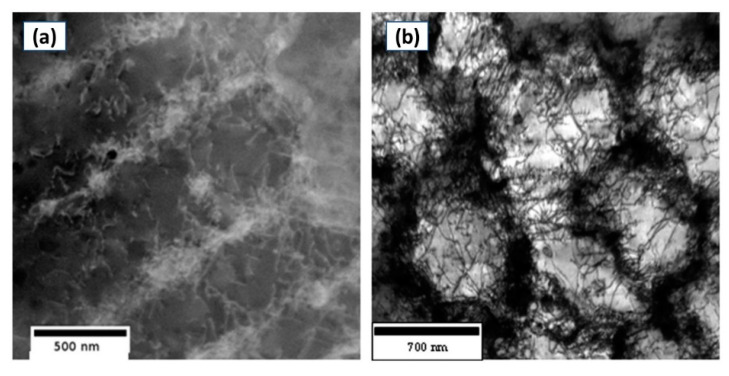
TEM image illustrating the dislocation network of the as-printed IN718 in: (**a**) columnar microstructure; (**b**) cellular microstructure [7]. Copyright 2017, Elsevier E. V.

Vickers microhardness testing was performed for both the as-printed and heat-treated conditions (before and after aging treatment) to investigate the effect of variations in the homogenization and solution treatment holding times on the strengthening of the laser powder bed fused IN718. Also, the evolution of the grain aspect ratio in the five heat-treatment conditions was added to correlate the mechanical properties with the microstructure. As can be seen in Figure 15, changes in the heat-treatment holding times have a significant impact on the hardness measurements. Before aging treatment, it can be seen that the as-printed condition exhibited higher hardness than any of the heat-treated conditions. This is attributed to the substructure/strain strengthening mechanism. It is well recognized that the microstructure of as-printed conditions contains a high density of dislocation tangles due to the fast melting and solidification during the printing process as reported by Cao et al. [36] and Tucho et al. [7] and shown in Figure 16 [7]. The presence of such a dislocation network strengthens the IN718 material through the strain strengthening mechanism.

After applying the heat-treatments without aging, hardness measurements decreased by 6–29% of the as-printed condition depending on the variation in the treatment times, as shown in Figure 15. This reduction in the hardness can be explained by the annihilation of some dislocations and other lattice defects with increasing the holding time. However, among the heat-treated conditions, the 1 h homogenized condition, HS1 and HS2, exhibited a slight reduction in the hardness, 6% and 8.6%, respectively, of the as-printed conditions suggesting that the 1 h treatment was not enough to eliminate the substructure and dislocation network. On the other hand, after prolonged homogenization treatment, a significant reduction in the hardness in relation to the as-printed, HS1 and HS2 conditions is observed. This can be explained by the longer holding time of the homogenization treatment (4 h) in HS3 results in promoting the recrystallization followed by grain growth after the longer homogenization time (7 h) in the HS4 and HS5 conditions. These findings are consistent with the microstructure results and the aspect ratio development as shown in Figure 15.

Generally, under the same aging conditions, the hardness of the as-printed samples significantly increased by 51–72% depending on the variation in the treatment time. This is because of the precipitation of the strengthening phases, γ′ and γ″, after the aging treatment. As can be seen in Figure 15, the HSA1 and HSA2 conditions exhibited the highest hardness among the five treatment conditions, which is due to the combined effects of precipitate strengthening (γ′ and γ″) and substructure strengthening (dislocation network) mechanisms. Moreover, by recalling the lattice parameter calculations of the γ-matrix after HSA1 and HSA2 treatments, significant back diffusion of the strengthening elements is observed in relation to the other treatment conditions. Thus, more precipitation of γ′ and γ″ are expected after the HSA1 and HSA2, which consequently strengthens the material. After the HSA3, HSA4 and HSA5 conditions, however, lower hardness is observed in relation to the other heat-treatment conditions due to the combination of grain growth accompanied by stress relief and precipitation of carbides. As confirmed by the microstructure results, the latter process consumed Nb and Ti at the expense of the γ″ and γ′ precipitates. Based on the above analysis, it can be concluded that the precipitation hardening is the dominant strengthening mechanism of the laser powder bed fused IN718 superalloy after the application of the HSA3, HSA4 and HSA5 conditions.

## 4. Conclusions

In the present study, the influence of homogenization and solution treatment times on the microstructure, phases precipitation and/or dissolution, texture evolution and Vickers microhardness of IN718 alloy processed by laser powder bed fusion process has been investigated. The main observations can be summarized as follows:

- The as-printed microstructure of IN718 consists of elongated grain morphologies oriented parallel to the building direction and a very fine dendritic substructure that epitaxially grows across several solidified layers. Such a microstructure is completely different from that encountered in the cast and wrought materials. Thus, the kinetics during the heat-treatment of the laser powder bed fused IN718 parts are different from its cast or wrought forms.
- Homogenization holding time has a significant impact on the microstructure, precipitates, crystallographic orientation and mechanical properties of the laser powder bed fused IN718. However, the 1 h homogenization treatment at 1080 °C is not enough to change the as-printed texture and grain structure. Also, Laves phase and inter-dendritic segregates are only partially dissolved after this treatment.
- Complete recrystallization and more dissolution of the (Nb, Ti)-rich segregates along with Laves phase are achieved after 4 h homogenization treatment at 1080 °C. Nevertheless, more carbide particles are formed with 4h homogenization treatment. Further increase in the homogenization time (7h) results in grain growth and carbides coarsening.
- The increase in the solution time at 980 °C does not significantly affect the grain structure and material texture. However, the amount of δ-phase is directly proportional to the duration of the solution heat-treatment.
- After post-treatments, the hardness significantly increases by 51–72% depending on the treatment time because of the precipitation of γ′ and γ″. Among the heat-treated conditions, the 1 h homogenization treatment conditions exhibit the highest hardness which decreases by increasing soaking time.
- Both substructure/strain (dislocation network) strengthening and precipitation hardening (γ′ and γ″) are the strengthening mechanisms in the 1 h homogenized treatment condition, while in the 4 h and 7 h homogenized conditions, mainly precipitation hardening strengthening mechanism is attained.

A systematic investigation will continue focusing on the elevated-temperature mechanical behavior of the laser powder bed fused IN718 following the same post-treatment window scheme. Furthermore, analyzing the fracture surface and correlating the high-temperature mechanical properties with the initial microstructure evolution is in progress.

## Figures and Tables

**Table 1:** Nominal chemical composition of gas atomized IN718 powder.

Element	Ni	Cr	Nb	Mo	Ti	Al	Fe + Traces
**wt.%**	49.19	19.04	4.92	2.70	1.08	0.33	Bal.

**Table 2:** Designations of specimens and the details of the corresponding post-treatment conditions.

Designation	Homogenization Heat-Treatment (H)	Solution Heat-Treatment (S)	Aging Heat-Treatment (A)
**As-printed**	None	None	None
**HSA1**	1080 °C for 1 h/AC*	980 °C for 15 min/AC	720 °C/8 h/FC** at 55 °C/h to 620 °C + 620 °C/8 h/AC
**HSA2**	1080 °C for 1 h/AC	980 °C for 1 h/AC	
**HSA3**	1080 °C for 4 h/AC	980 °C for 37.5 min/AC	
**HSA4**	1080 °C for 7 h/AC	980 °C for 15 min/AC	
**HSA5**	1080 °C for 7 h/AC	980 °C for 1 h/AC	

*AC: air cooling, **FC: furnace cooling.

**Table 3:** EDS spot analysis results of the as-printed condition in Figure 5 (wt.%).

Spectrum	Phase	Fe	Ni	Cr	Nb	Ti	Mo	Al
1, 2	Laves	Bal.	43.55 ± 3.9	15.83 ± 1.25	14.45 ± 6.53	1.85 ± 0.71	3.62 ± 0.01	0.44 ± 0.08
3	Matrix	Bal.	50.03 ± 0.46	18.51 ± 0.19	3.88 ± 0.01	0.79 ± 0.03	3.22 ± 0.07	0.56 ± 0.11

**Table 4:** EDS spot analysis results of the heat-treated conditions in Figure 11 (wt.%).

Spectrum	Phase	Fe	Ni	Cr	Nb	Ti	Mo	Al
1	Matrix	Bal.	50.02 ± 0.43	18.25 ± 0.21	5.59 ± 0.15	0.98 ± 0.08	3.38 ± 0.11	0.48 ± 0.03
2	Laves	Bal.	44.82	13.77	14.32	1.68	2.72	0.43
3	Matrix	Bal.	49.70 ± 0.47	18.28 ± 0.19	5.46 ± 0.31	0.96 ± 0.05	3.36 ± 0.17	0.50 ± 0.02
4	δ- phase	Bal.	53.70	10.94	15.44	1.57	2.40	0.30
5	Carbides	Bal.	14.52	6.44	52.17	5.80	-	-
